# Supplementary figures and images for: Identification of a large anion channel required for digestive vacuole acidification and amino acid export in Plasmodium falciparum
Source: PLoS Biol. 2025 May 30;23(5):e3003202. doi: 10.1371/journal.pbio.3003202 (PMC12158007; doi:10.1371/journal.pbio.3003202)

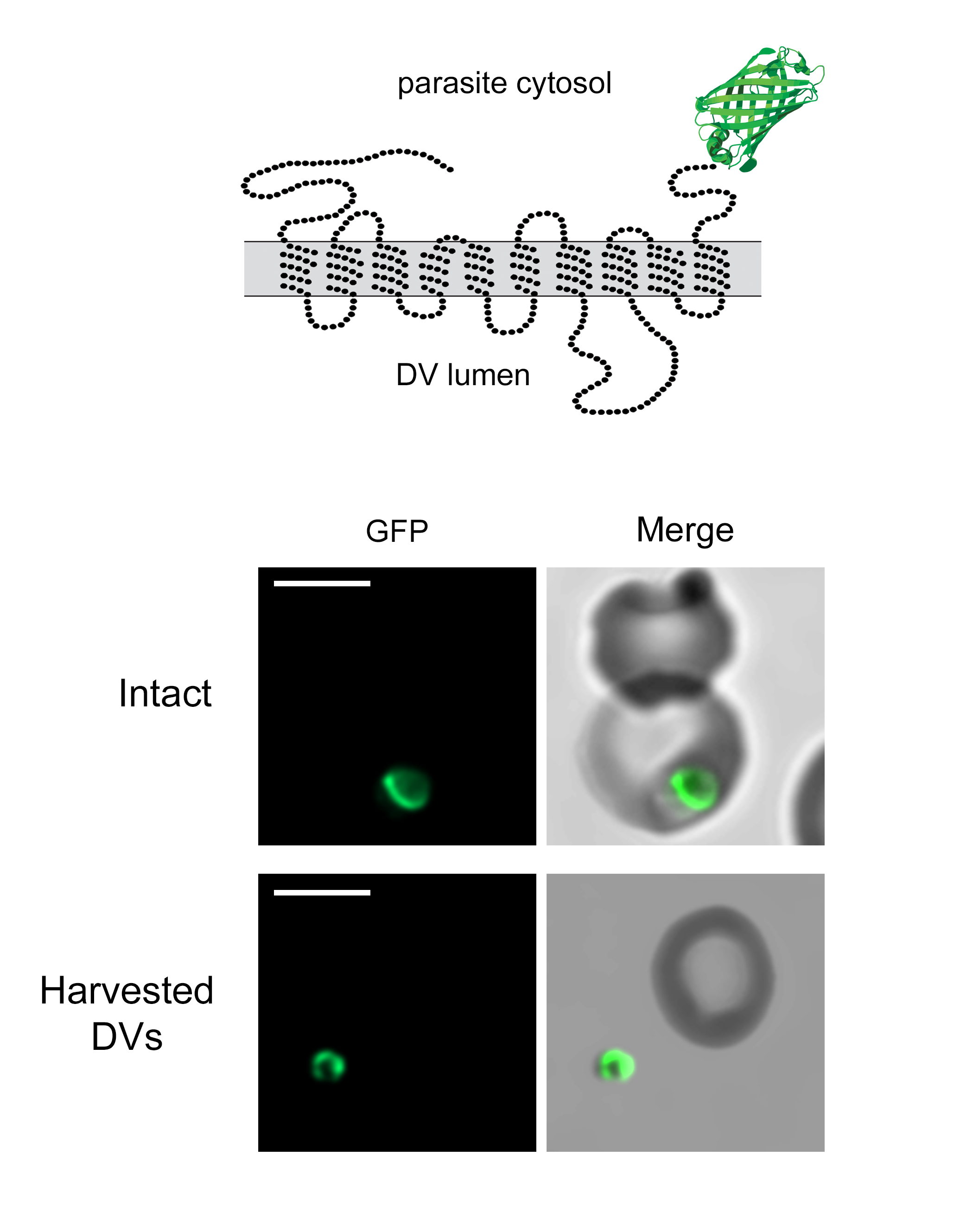

Supplement: S1 Fig — Schematic shows PfCRT transmembrane topology with residues indicated by individual dots (adapted from [65]); GFP (green ribbon) is predicted to localize in parasite cytosol. Confocal fluorescence images showing intact infected erythrocytes or isolated DV expressing GFP-tagged PfCRT, a DV membrane protein marker. Excitation/emission: 485 ± 20 nm/530 ± 25 nm. Scale bars, 5 µm. (TIF) [file pbio.3003202.s001.tif]

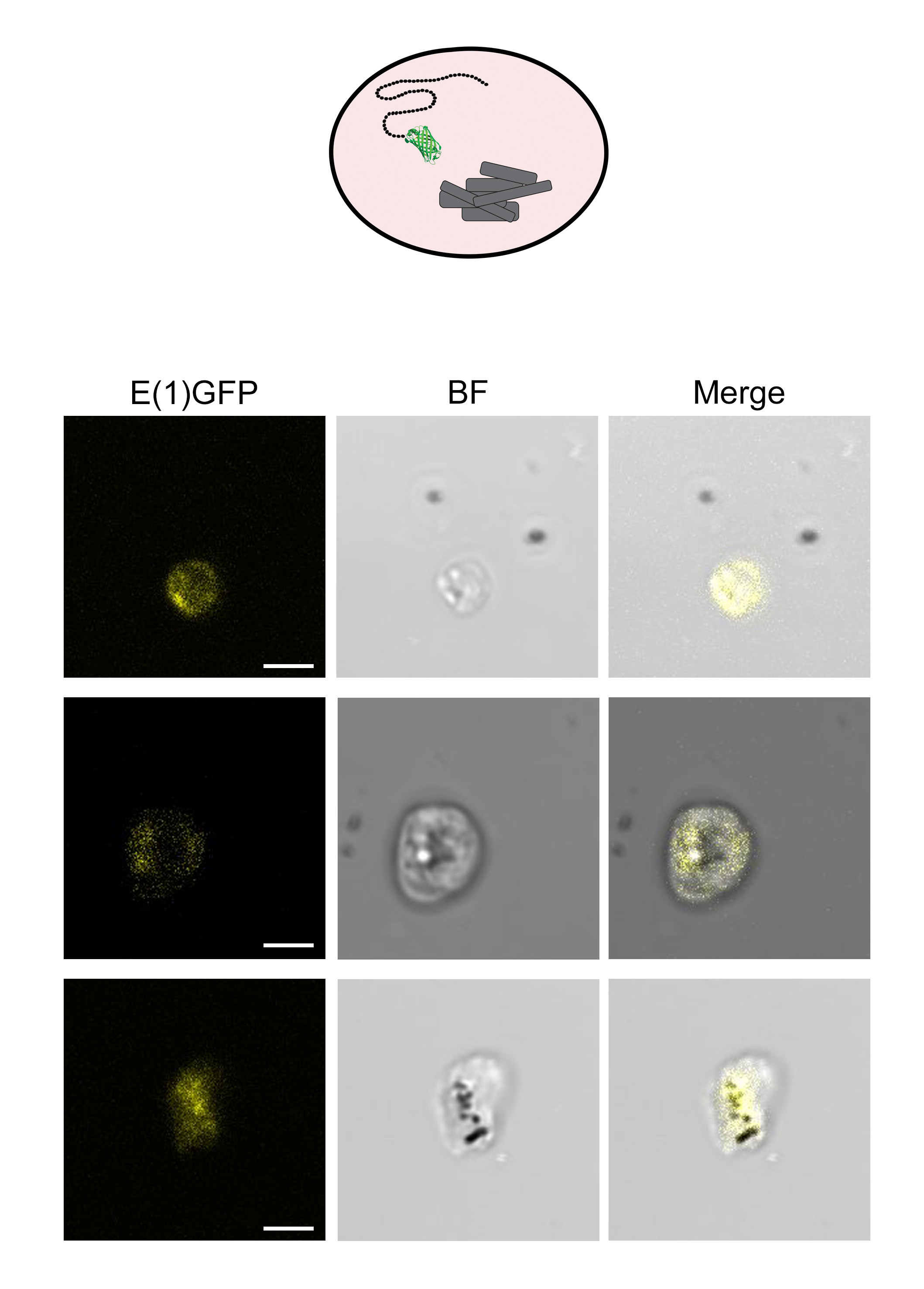

Supplement: S2 Fig — Confocal fluorescence, brightfield and merge images showing three separate DVs, harvested the plasmepsin II-E(1)GFP reporter line. Retention of this soluble protein indicates preserved DV integrity. Scale bars, 2 µm. (TIF) [file pbio.3003202.s002.tif]

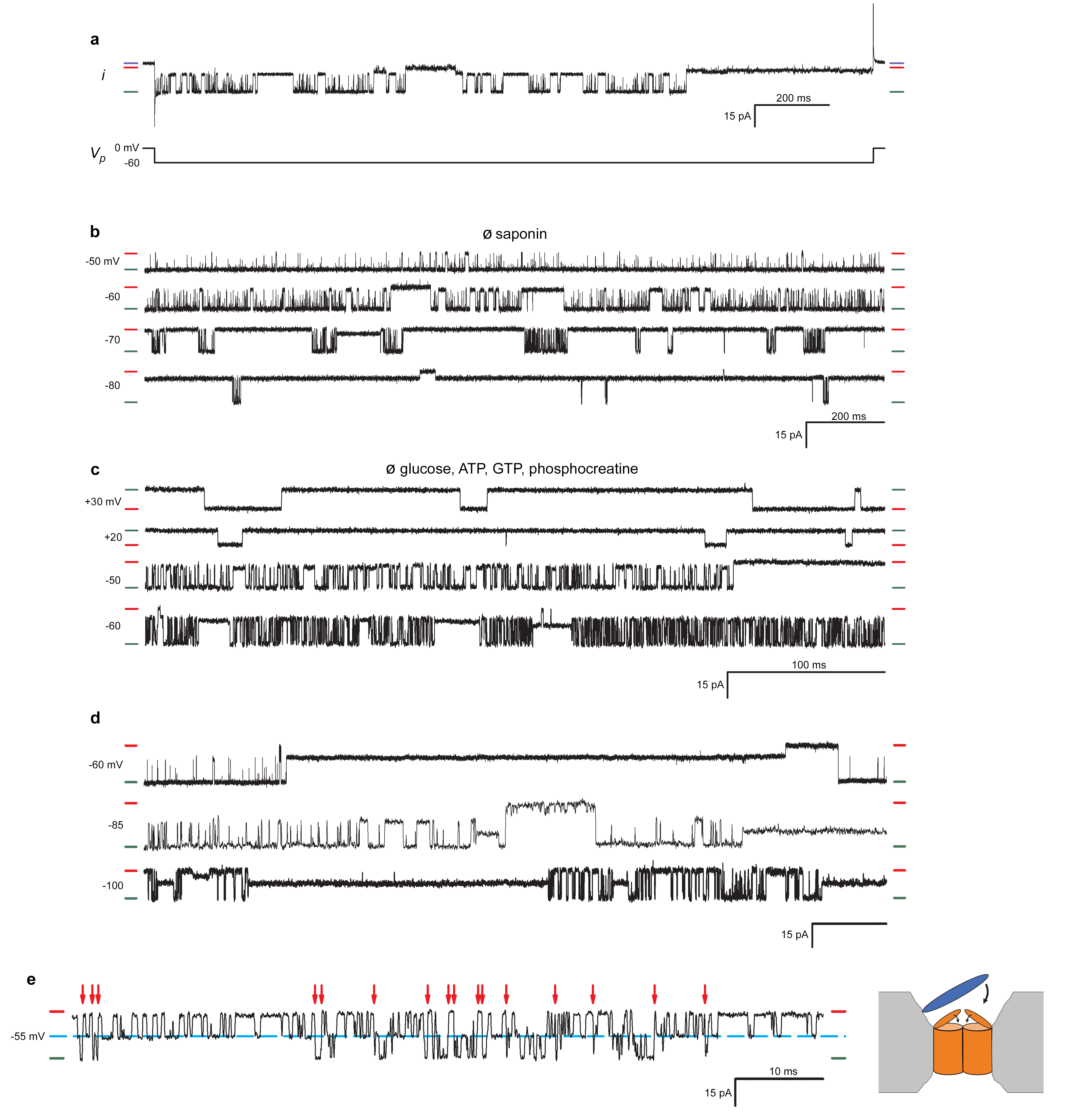

Supplement: S3 Fig — (A) Single channel recordings at indicated Vp using DV harvest without saponin; pipette and bath solution: Buffer A with WOS. Unchanged channel activity excludes artifacts associated with detergent exposure. Closed and open channel levels, red and green dashes. (B) Recordings without glucose, ATP, GTP, or phosphocreatine in the bath or pipette solutions (Buffer A). Channel activity does not require external energy sources. (C) Complex channel gating with multiple subconductance levels, apparent as current levels intermediate between the fully closed and fully open levels marked by red and green dashes. Buffer A with WOS in pipette and bath. Horizontal scale bar: 475.4, 17.0, and 46.8 ms (top to bottom traces, respectively). We excluded independent small-conductance channels in these recordings because of clear interconversions between all current levels; such interconversions are not observed with independent channels. (D) Single channel recording showing a functional channel dimer, recorded with Buffer A plus WOS in pipette and bath. Two separate channels in the patch are excluded as there are frequent transitions between both pores closed and both pores open, as marked with red down arrows. The current level corresponding to a single open pore is marked with a dashed blue line. Schematic at right shows a possible structural model for this behavior, where closing of the master gate (blue) could produce the observed double transitions (red down arrow); individual pore gates (orange) account for transitions to and from the dashed blue line. The underlying data can be found at https://doi.org/10.5281/zenodo.15305314. (TIF) [file pbio.3003202.s003.tif]

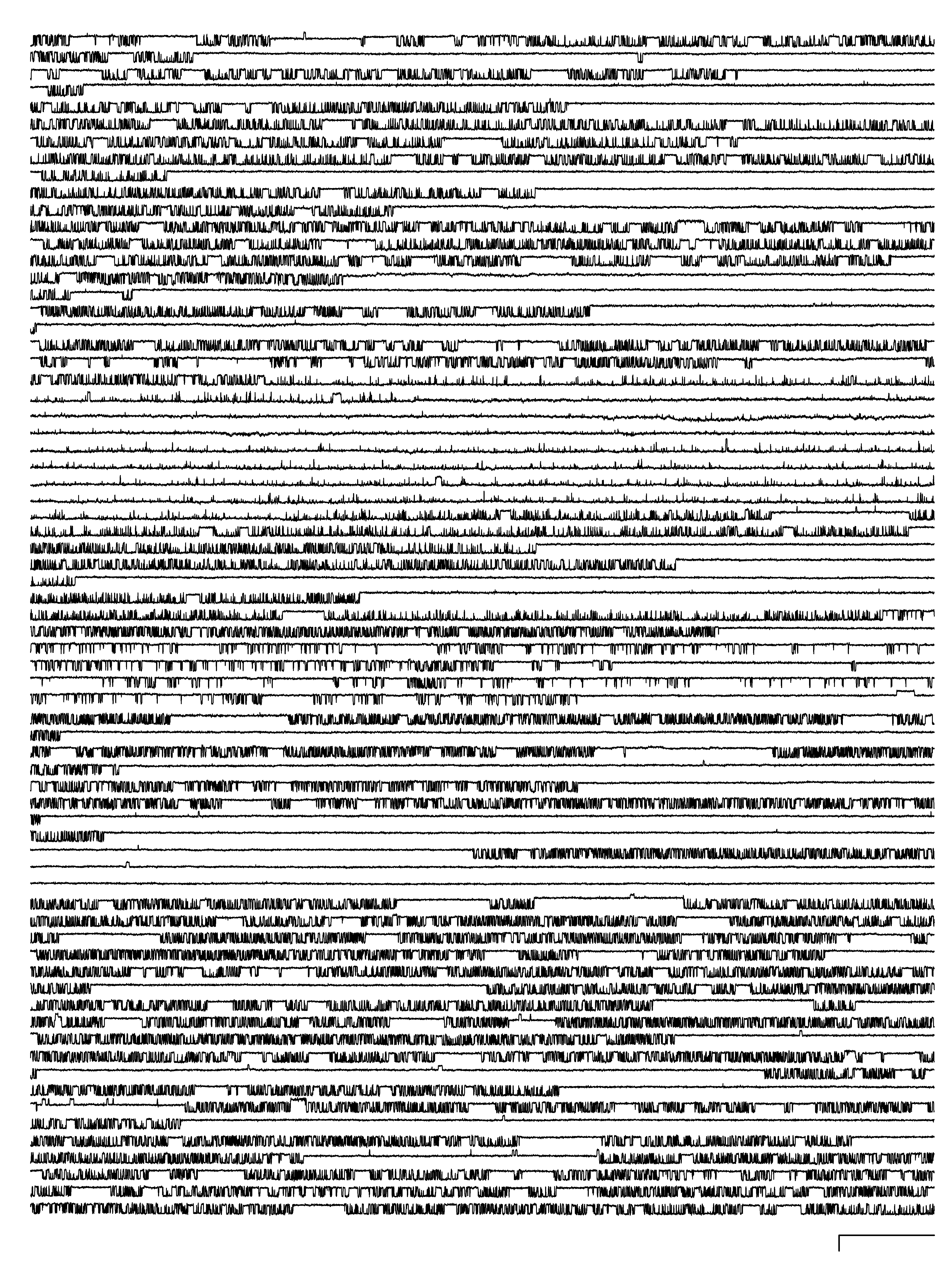

Supplement: S4 Fig — Consecutive 500 ms traces recorded with Buffer A in bath and pipette. Upgoing transitions reflect channel closings. Notice the bursts of complex gating mixed with periods of long closings and openings; subconductance levels are also apparent in many sweeps. Vp = −60 mV. Scale bar at bottom right, 25 pA/50 ms. The underlying data can be found at https://doi.org/10.5281/zenodo.15305314. (TIF) [file pbio.3003202.s004.TIF]

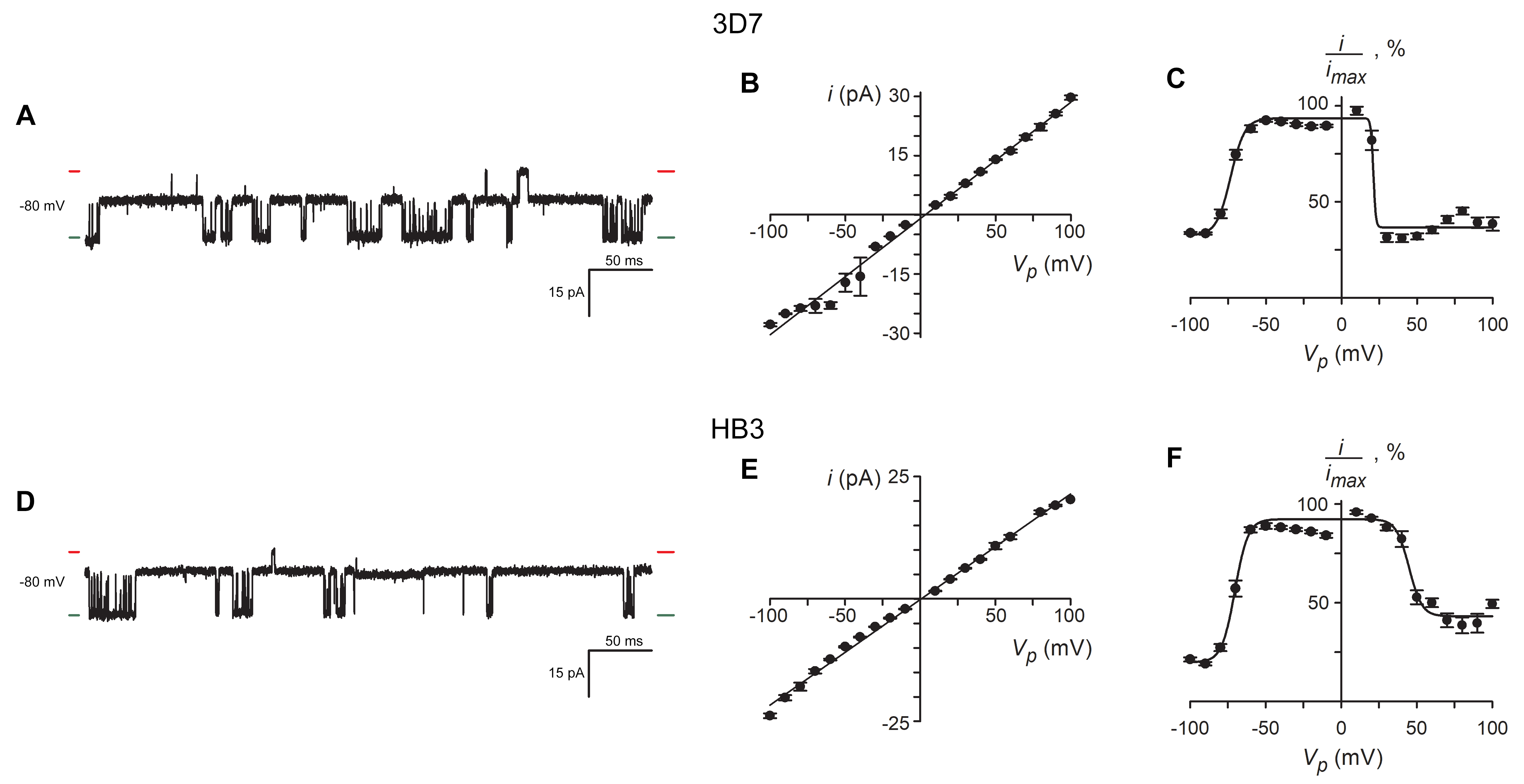

Supplement: S5 Fig — (A, D) Single channel recordings on DVs from 3D7 and HB3 parasites, respectively, using Buffer A plus WOS. Notice the conserved single-channel amplitudes, gating, and subconductance levels. Vp, −80 mV for both traces. Closed and open channel levels, red and green dashes. (B, E) Current-voltage relationships for 3D7 and HB3 channel molecules. (C, F) Open probabilities at imposed Vp for 3D7 and HB3 channels. Solid lines, best fit to Eq. 2. There is greater molecule-to-molecule variation within a single strain than between these geographically divergent strains with distinct antimalarial susceptibilities. The underlying data can be found at https://doi.org/10.5281/zenodo.15305314. (TIF) [file pbio.3003202.s005.tif]

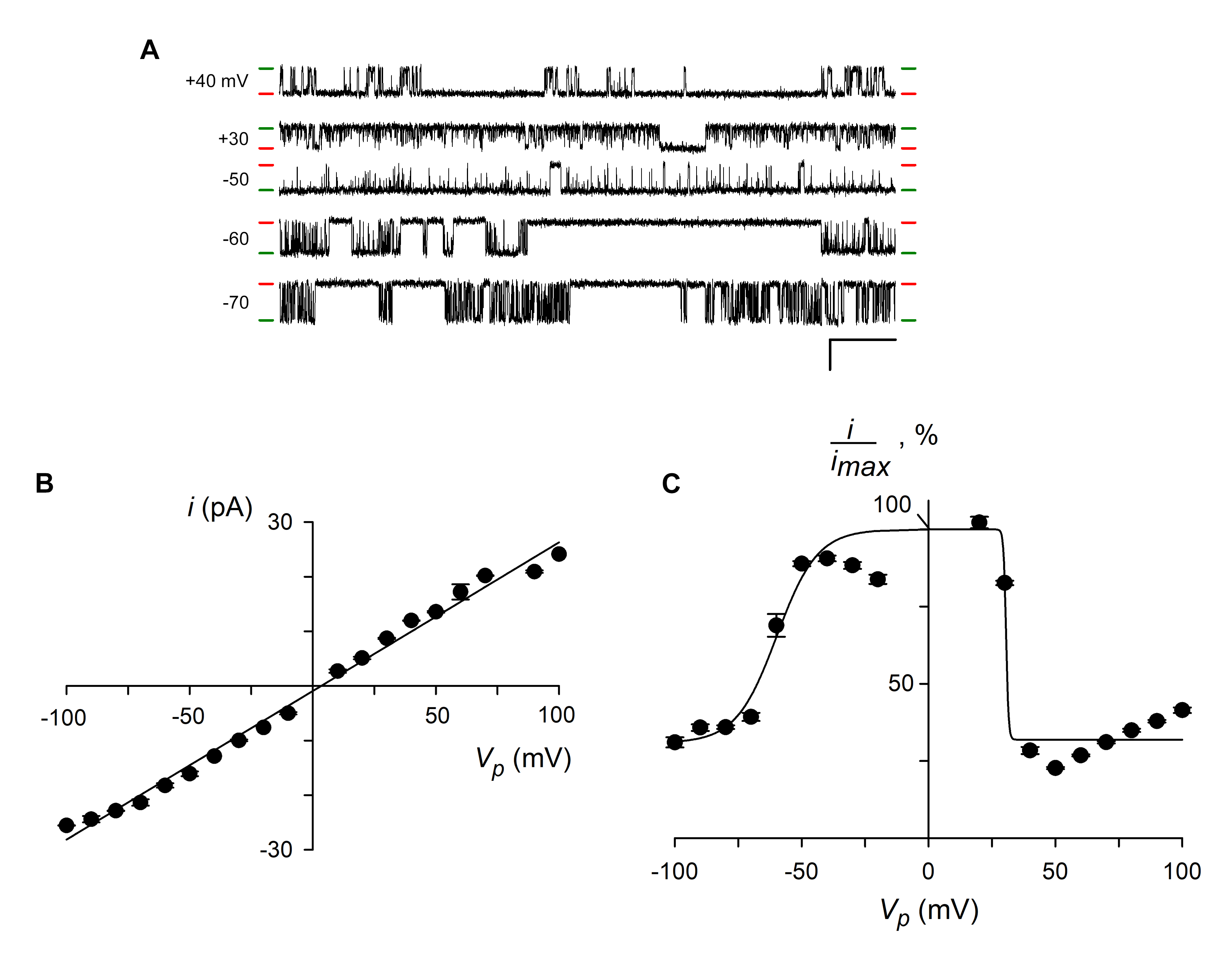

Supplement: S6 Fig — (A) Single BVAC recordings with Buffer A in pipette and Buffer L in bath. Vp as indicated; closed and open channel current levels, red and green dashes; scale bar, 10 pA/50 ms. (B) Current–voltage (i–V) relationship. The single-channel conductance, 272 pS, is not significantly affected by luminal alkalization. (C) Open probability vs. Vp plot. Solid line, best fit to Eq. 2. The underlying data can be found at https://doi.org/10.5281/zenodo.15305314. (TIF) [file pbio.3003202.s006.tif]

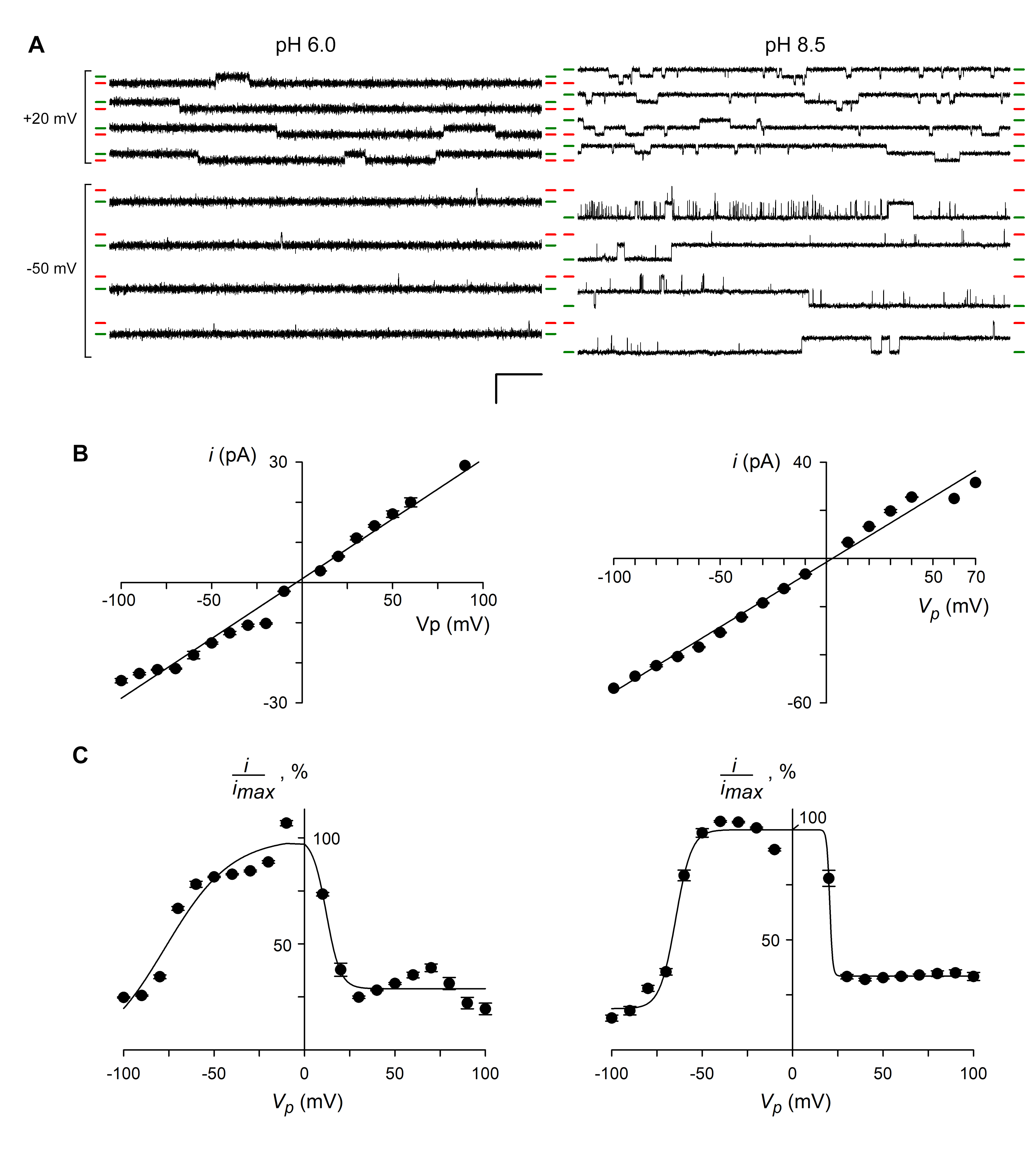

Supplement: S7 Fig — (A) Single BVAC recordings with Buffer A in pipette after adjusting pH to 6.0 or 8.5 pipette (left and right traces); Buffer A in bath. Notice reduced gating at pH 6.0 and functional dimerization at pH 8.5. Vp as indicated; closed and open channel current levels, red and green dashes; scale bar, 20 pA/50 ms. (B) Current–voltage (i–V) relationships with pipette solutions at pH 6.0 and 8.5 (left and right panels). Note the increased conductance of 540 pS at pH 8.5 at the cytosolic channel face. (C) Open probability vs. Vp plots for these conditions. The underlying data can be found at https://doi.org/10.5281/zenodo.15305314. (TIF) [file pbio.3003202.s007.tif]

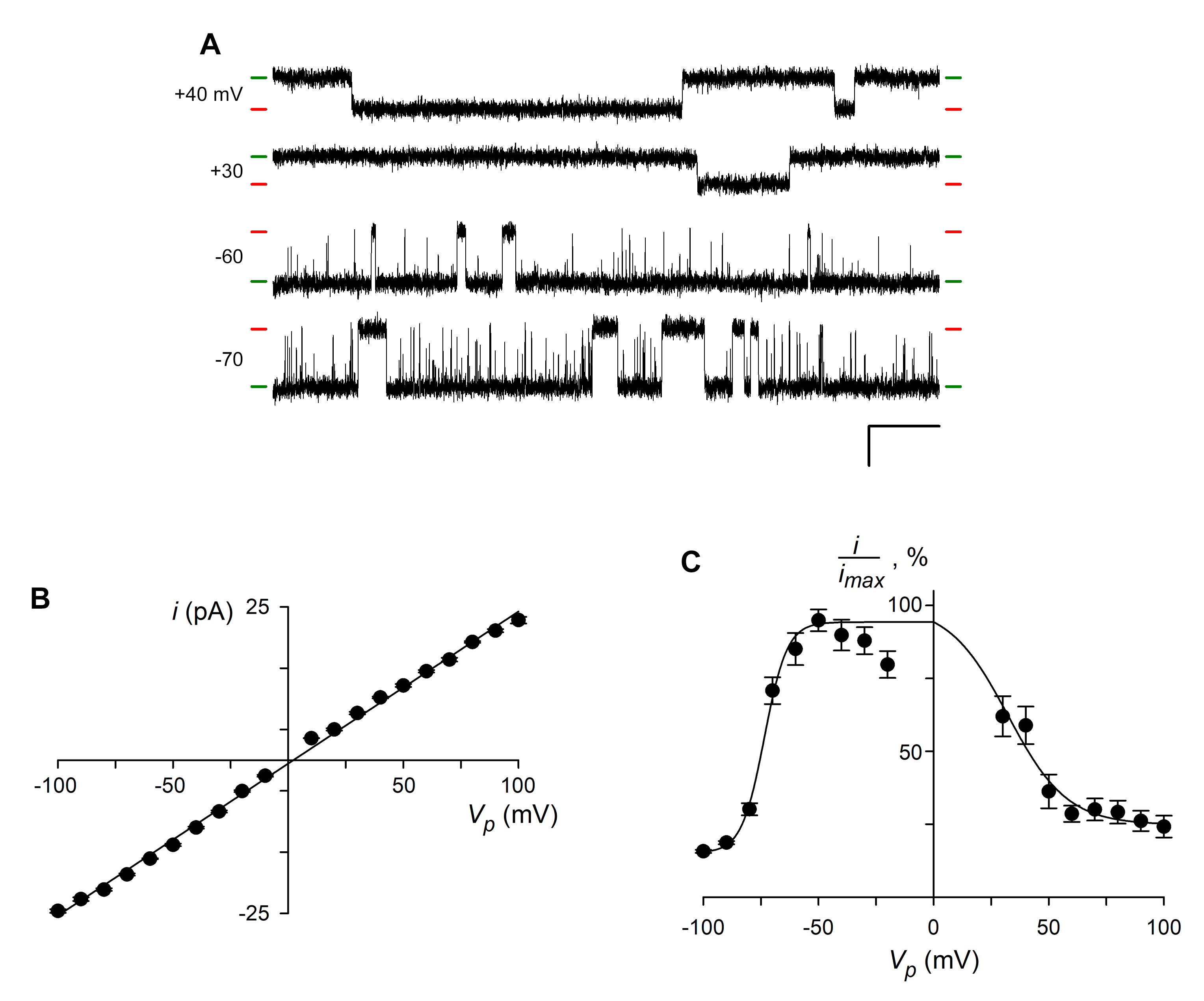

Supplement: S8 Fig — (A) BVAC recordings with Buffer M in pipette and bath. Vp as indicated; closed and open channel current levels, red and green dashes; scale bar, 10 pA/50 ms. (B) Current–voltage (i–V) relationship. (C) Open probability vs. Vp plot. Solid line, best fit to Eq. 2. The underlying data can be found at https://doi.org/10.5281/zenodo.15305314. (TIF) [file pbio.3003202.s008.tif]

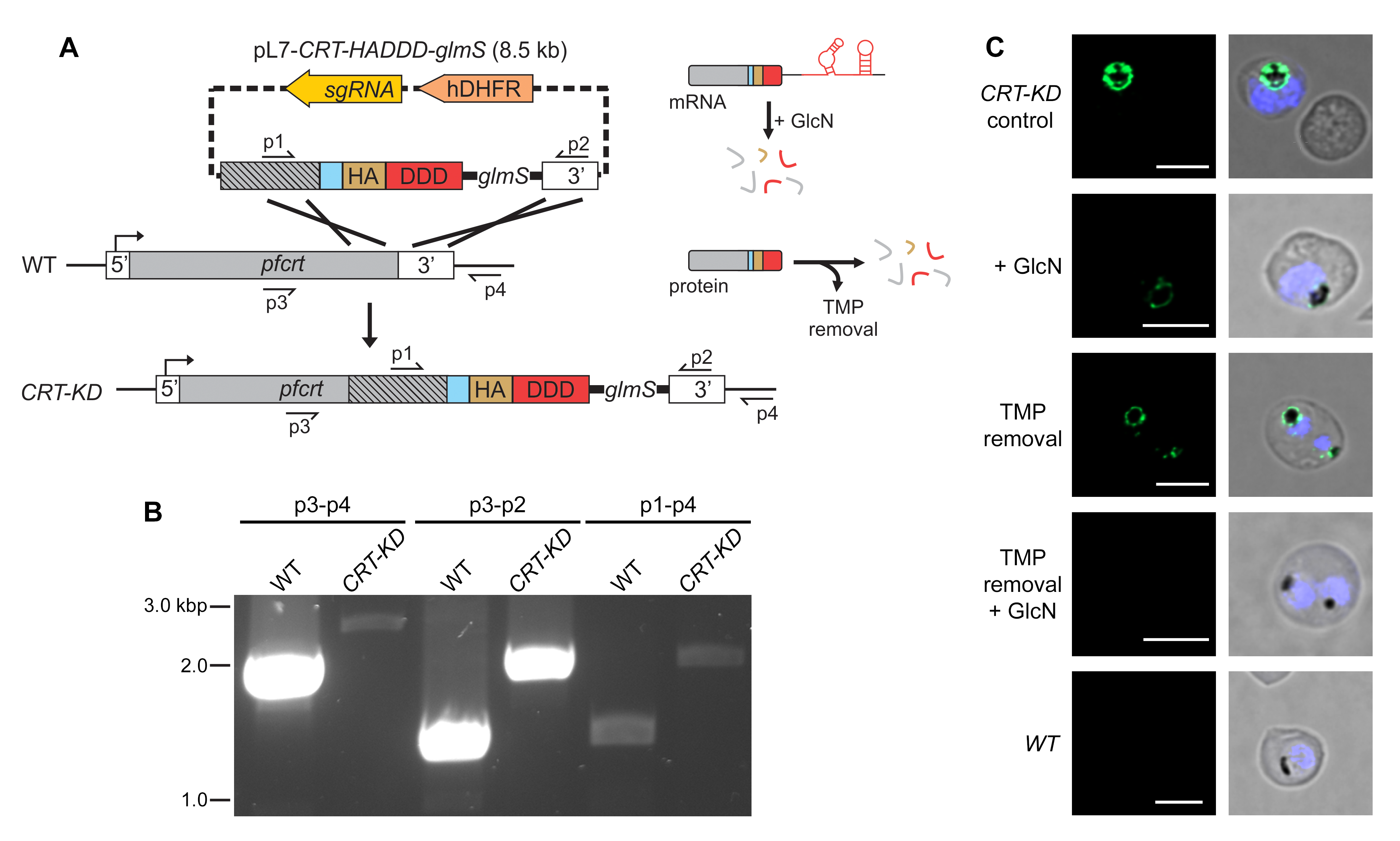

Supplement: S9 Fig — (A) Schematic showing CRISPR/Cas9 strategy for the engineered CRT-KD line. The glmS riboswitch permits conditional regulation of transcript abundance through GlcN-dependent mRNA degradation while DDD allows post-translational regulation through protein denaturation upon TMP removal. (B) Ethidium-stained gel showing PCR integration checks with primer positions as indicated in panel (A). Primers are listed in S1 Table. Expected amplicon sizes for each primer pair (in bp): p3-p4, 1856 (WT) and 2422 (CRT-KD); p3-p2, 1276 (WT) and 1833 (CRT-KD); p1-p4, 1304 (WT) and 1870 (CRT-KD). (C) Indirect immunofluorescence images of trophozoite-stage CRT-KD and WT parasites under indicated conditions and probed with anti-HA antibody. The CRT-KD control was grown with TMP and without GlcN. WT was not recognized, indicating antibody specificity. Scale bars, 5 µm. (TIF) [file pbio.3003202.s009.tif]

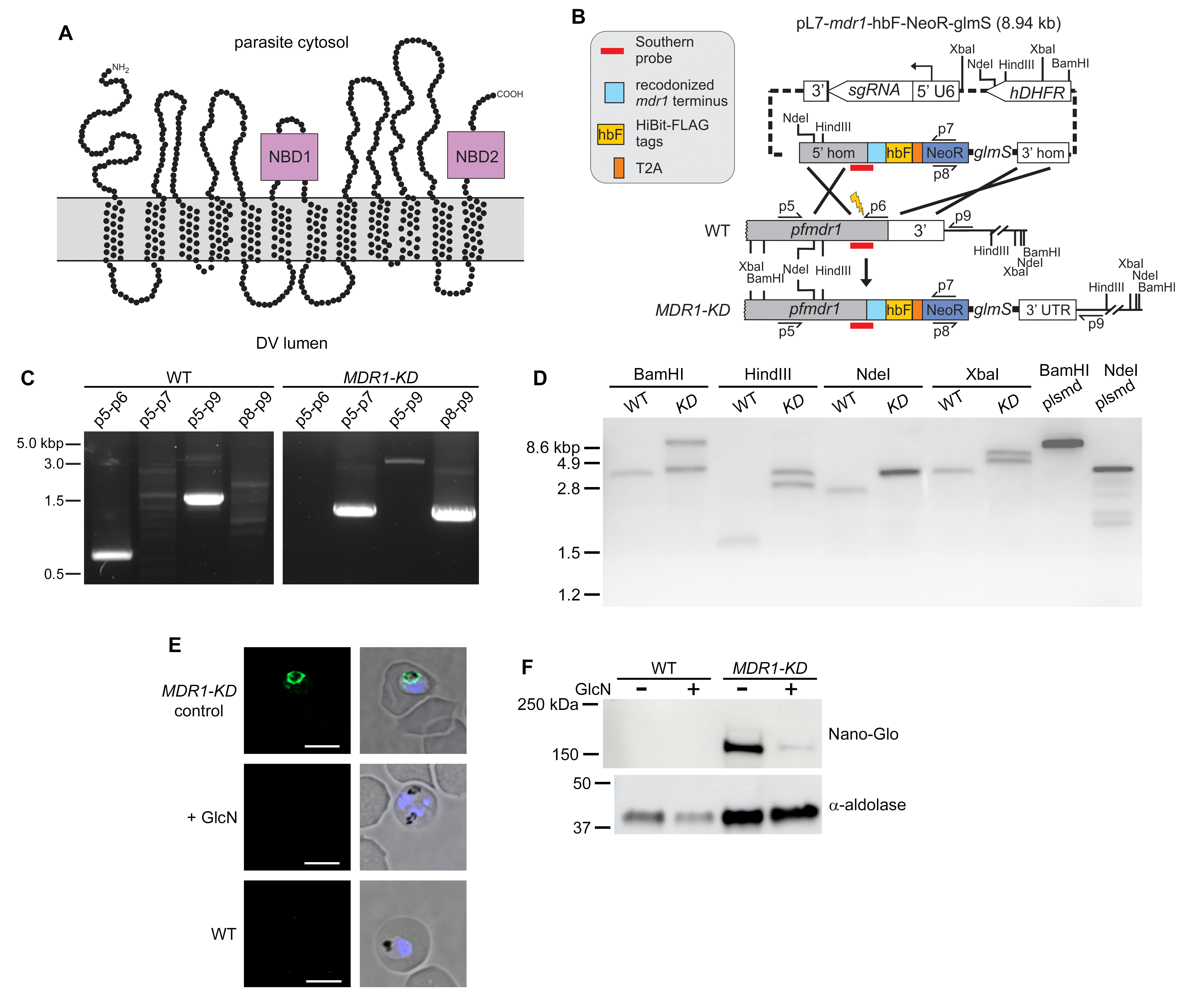

Supplement: S10 Fig — (A) PfMDR1 transmembrane topology showing 12 predicted transmembrane domains and 2 nucleotide-binding domains (NBD) for ATP binding (adapted from ref. [66]). (B) CRISPR/Cas9 strategy for the engineered MDR1-KD line showing the glmS riboswitch. C-terminal epitope tags are followed by a T2A ribosome skip peptide sequence and a neomycin resistance gene (NeoR) to facilitate selection-linked integration. The Southern probe hybridizes to both WT and MDR1-KD sequences; restriction sites reflect expected Southern blot cleavage products. (C) Gel showing PCR integration checks for indicated parasites and primer pairs (panel (B), S1 Table). Expected amplicon sizes (bp): p5-p6, 756 (WT only); p5-p7, 1291 (MDR1-KD only); p5-p9, 1697 (WT) and 2803 (MDR1-KD); p8-p9, 1532 (MDR1-KD only). (D) Southern blot for MDR1-KD clone (KD) and WT parent, establishing a single pfmdr1 copy in the Dd2 wild-type parental line (WT) and complete replacement with the conditional knockdown cassette in MDR1-KD. Expected digestion product sizes (bp): BamH1, 3444 (WT), 4591 (MDR1-KD), 8935 (plasmid); HindIII, 1700 (WT), 2838 (MDR1-KD); NdeI, 2507 (WT), 3654 (MDR1-KD), 3660 (plasmid); XbaI, 3607 (WT), 4754 (MDR1-KD). Doublets seen in the MDR1-KD lanes reflect the genomic and retained plasmid in the transfectant clone (lower and upper bands, respectively). (E) Anti-FLAG immunofluorescence images of MDR1-KD grown without and with GlcN, establishing localization at the DV membrane and knockdown. The WT parent is not recognized. Scale bars, 5 µm. (F) Nano-Glo HiBit blot showing PfMDR1 knockdown in MDR1-KD with GlcN. The wild-type negative control (WT, left) and aldolase loading control (bottom) are included. (TIF) [file pbio.3003202.s010.tif]

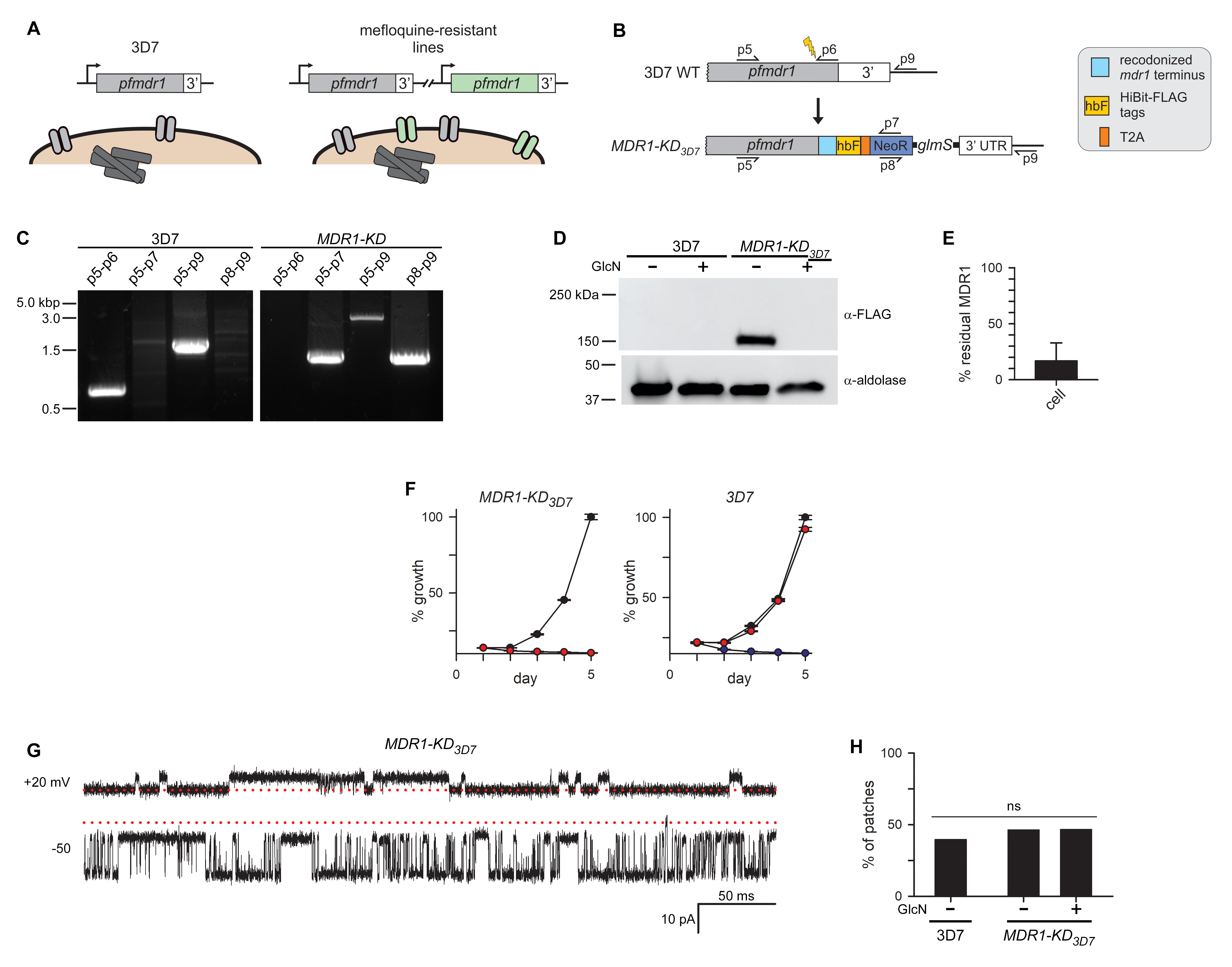

Supplement: S11 Fig — (A) Prevailing model for how pfmdr1 gene duplication increases transporter abundance and produces resistance to antimalarials including mefloquine. Schematic shows 3D7 with one copy and a mefloquine-resistant line with two copies. Bottom, PfMDR1 is shown on the DV membrane and is color-coded to match gene color in the ribbon. (B) Ribbon schematic showing the 3D7 wild-type (WT) and its engineered daughter, MDR1-KD3D7. Modifications and primer positions are indicated. (C) PCR integration checks for indicated parasites and primer pairs (panel (B), S1 Table). Expected amplicon sizes (bp): p5-p6, 756 (3D7 only); p5-p7, 1291 (MDR1-KD3D7 only); p5-p9, 1697 (3D7) and 2803 (MDR1-KD); p8-p9, 1532 (MDR1-KD3D7 only). (D) Anti-FLAG immunoblot showing conditional knockdown of PfMDR1 in MDR1-KD3D7 total cell membranes upon GlcN treatment. The wild-type control and aldolase loading controls are included. (E) Mean ± S.E.M. % residual PfMDR1 after knockdown in MDR1-KD3D7 total membranes (cell), quantified using anti-FLAG immunoblots. (F) Mean ± S.E.M. expansion of indicated parasites over 5 days. Black circles, control medium; red circles, GlcN pulse; blue circles, chloroquine control (3D7 only). P = 0.01 for GlcN pulse in MDR1-KD3D7 but n.s. for 3D7. (G) Single channel recordings on MDR1-KD3D7 after PfMDR1 knockdown with GlcN. Buffer A in pipette and bath; Vp as indicated. Red dotted line, closed level. (H) % of patches with channels for indicated parasites and GlcN treatment. ns, no statistically significant difference between all tested arms. P ≥ 0.58 for pairwise comparisons. The underlying data can be found at https://doi.org/10.5281/zenodo.15305314. (TIF) [file pbio.3003202.s011.tif]

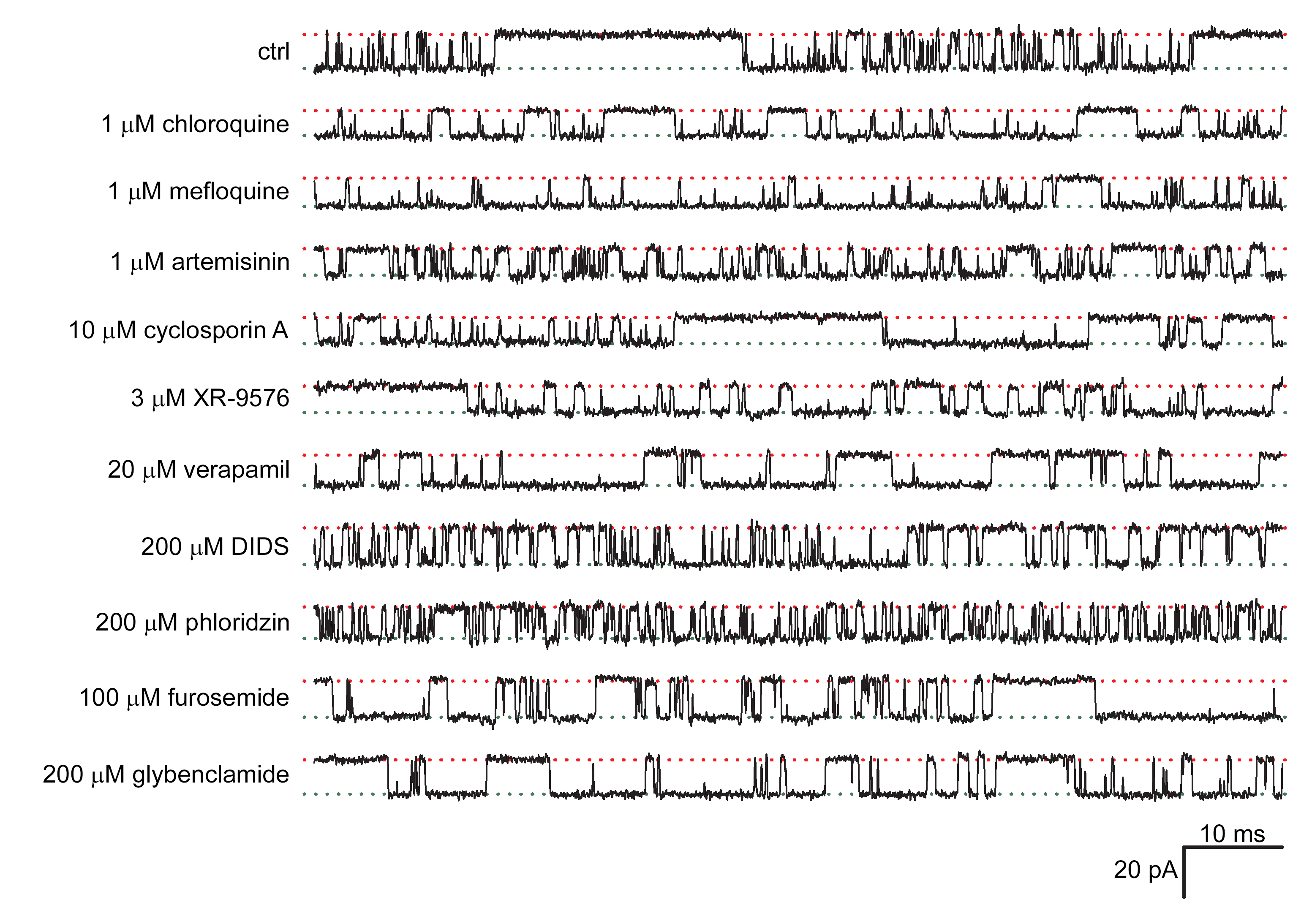

Supplement: S12 Fig — Single channel recordings on DV from Dd2 parasites with indicated inhibitor present in bath and pipette solutions (Buffer A plus WOS). Vp, −60 mV. Red and green dotted lines, closed and open channel levels. These inhibitors do not significantly alter BVAC gating. The underlying data can be found at https://doi.org/10.5281/zenodo.15305314. (TIF) [file pbio.3003202.s012.tif]

Fig. 3B  $\alpha$ -HA

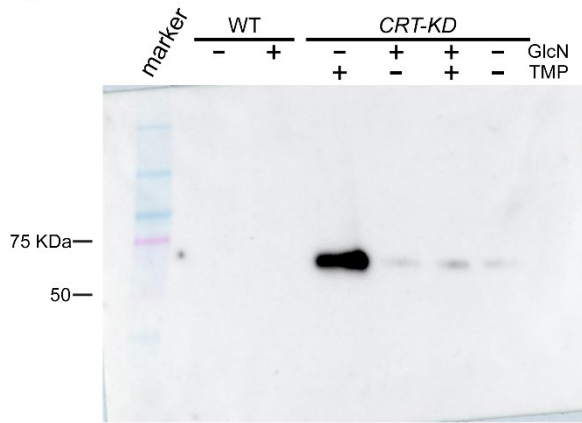

Fig. 3B  $\alpha$ -aldolase

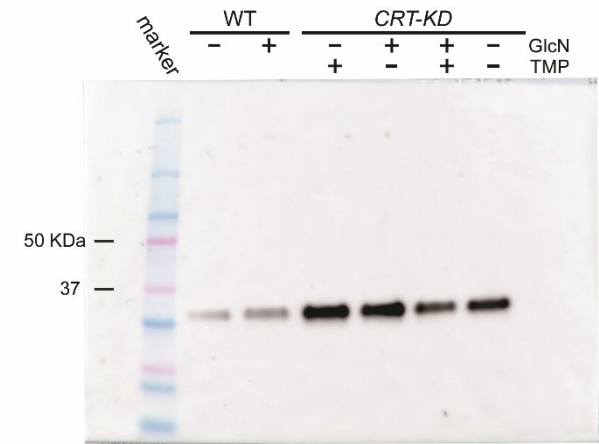

Fig. 3F

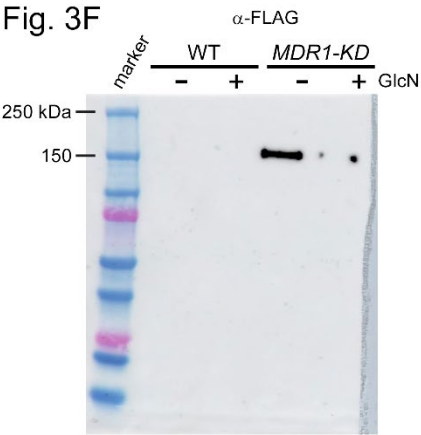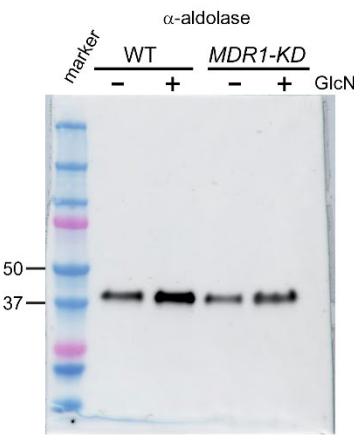

Fig. S9B

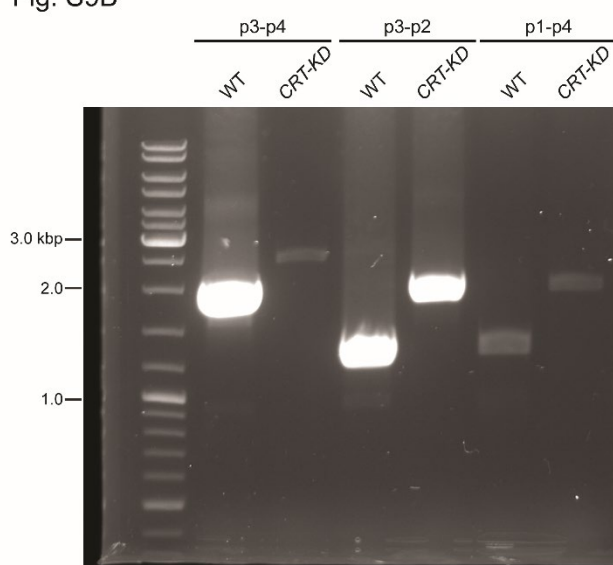

Fig. S10C

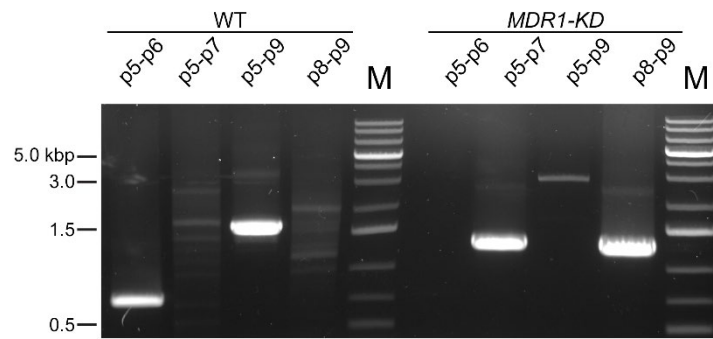

Fig. S10D

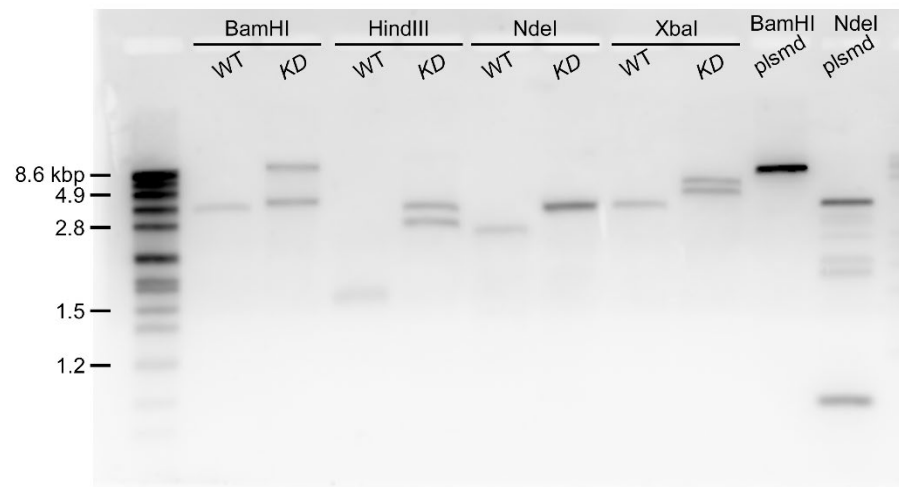

Fig. S10F

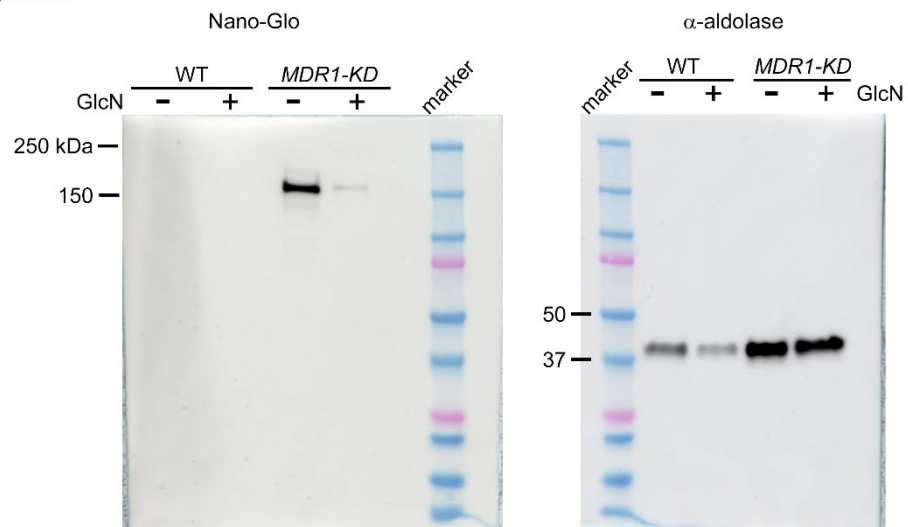

Fig. S11C

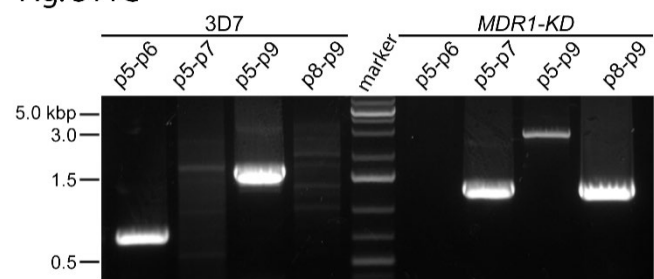

Fig. 11D

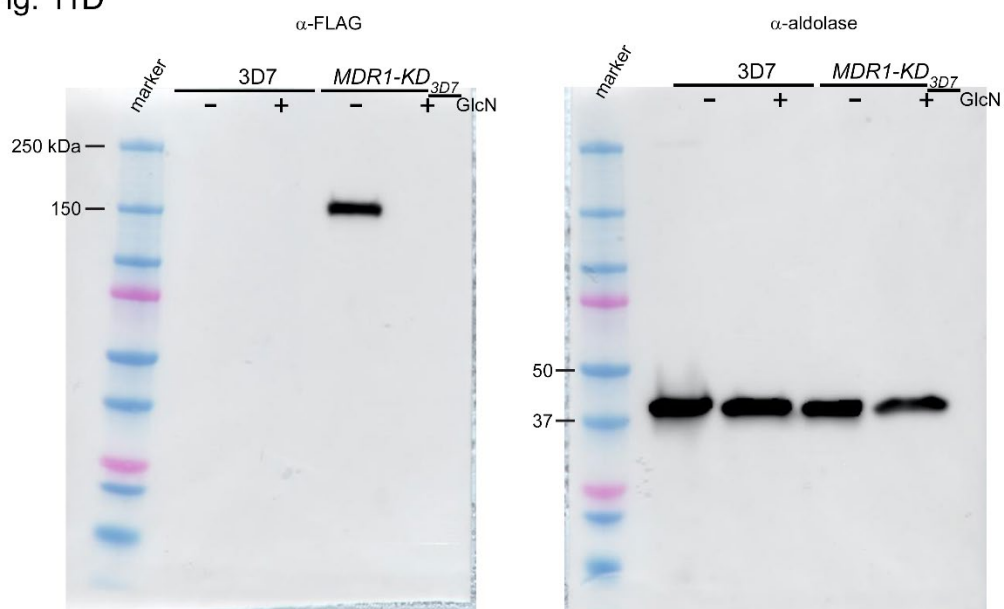

Supplement: S1 Raw Images — The raw data for Figs 1D–1F, 2A–2C, 3C, 3D, 3G–3I, 4, S5A–S5F, S6A–S6C, S7A–S7C, S8A–S8C, S11E–S11H, and S12 are available at https://doi.org/10.5281/zenodo.15305314. (PDF) [file pbio.3003202.s019.pdf]
