# Supplementary material for: Identification of a large anion channel required for digestive vacuole acidification and amino acid export in Plasmodium falciparum
Source: PLoS Biol. 2025 May 30;23(5):e3003202. doi: 10.1371/journal.pbio.3003202 (PMC12158007; doi:10.1371/journal.pbio.3003202)
Supplement: S2 Table — (DOCX) [file pbio.3003202.s014.docx]

| **S2 Table.**  Buffer compositions and additives for patch-clamp in this study. | |
| --- | --- |
| Buffer ID | Composition (in mM): |
| A | 70 NaCl, 70 KCl, 2.0 CaCl_2_, 2.5 MgCl_2_, 10 glucose, 10 HEPES, 10 MES, pH 7.4 with NaOH |
| B | 100 CaCl_2_, 10 HEPES, pH 7.4 with NaOH |
| C | 50 KCl, 2.0 CaCl_2_, 2.5 MgCl_2_, 10 HEPES, pH 7.4 with KOH |
| D | 280 sorbitol, 2.0 CaCl_2_, 2.5 MgCl_2_, 10 glucose, 10 HEPES, 10 MES, pH 7.4 with KOH |
| E | 140 K-glutamate, 2.0 CaCl_2_, 2.5 MgCl_2_, 10 glucose, 10 HEPES, 10 MES, pH 7.4 with KOH |
| F | 140 KCl, 2.0 CaCl_2_, 2.5 MgCl_2_, 10 glucose, 10 HEPES, 10 MES, pH 7.4 with KOH |
| G | 140 NaBr, 2.0 CaCl_2_, 2.5 MgCl_2_, 10 glucose, 10 HEPES, 10 MES, pH 7.4 with NaOH |
| H | 140 NaCl, 2.0 CaCl_2_, 2.5 MgCl_2_, 10 glucose, 10 HEPES, 10 MES, pH 7.4 with NaOH |
| I | 140 MgSO_4_, 2.0 CaCl_2_, 2.5 MgCl_2_, 10 glucose, 10 HEPES, 10 MES, pH 7.4 with NaOH |
| J | 140 KSCN, 2.0 CaCl_2_, 2.5 MgCl_2_, 10 glucose, 10 HEPES, 10 MES, pH 7.4 with KOH |
| K | 140 KH_2_PO_4_, 2.0 CaCl_2_, 2.5 MgCl_2_, 10 HEPES, pH 7.4 with KOH |
| L | Buffer A supplemented with 40 mM NH_4_Cl, 75 nM concanamycin A, pH 7.4 with NaOH |
| M | 70 NaCl, 70 KCl, 4.5 MgCl_2_, 10 glucose, 10 HEPES, 10 MES, pH 7.4 with NaOH |
|  |  |
| WOS additive | 1.0 Na_2_ATP, 0.3 Na_2_GTP, 8.8 Na_2_-phosphocreatine |
