# Supplementary material for: Identification of a large anion channel required for digestive vacuole acidification and amino acid export in Plasmodium falciparum
Source: PLoS Biol. 2025 May 30;23(5):e3003202. doi: 10.1371/journal.pbio.3003202 (PMC12158007; doi:10.1371/journal.pbio.3003202)
Supplement: Attachment — Submitted filename: Response to Reviewers 031425.docx [file pbio.3003202.s022.docx]

Reviewer #1:

*The manuscript describes the discovery of an anion conductance in the digestive vacuolar membrane of Plasmodium falciparum, the human malaria parasite. To achieve this, the authors developed an improved method for isolating intact digestive vacuoles, which presents significant challenges due to the sharp heme crystals within this organelle that can compromise membrane integrity. Additionally, the authors constructed particularly small patch-clamp pipettes, allowing them to successfully access the tiny food vacuoles for electrophysiological studies. These technical innovations are commendable and critical for the success of the study.*

*After overcoming these methodological obstacles, the authors characterized the anion conductance, demonstrating its voltage-dependent permeability to various inorganic and organic anions. Importantly, they utilized genetically engineered knockdown mutants to show that the anion conductance operates independently of PfCRT and PfMDR1, two well-characterized transporters of the digestive vacuolar membrane. Furthermore, the authors investigated the druggability of the anion conductance, employing antimalarial drugs and specific anion channel inhibitors. The study concludes with a proposed model suggesting that this anion conductance plays a key role in maintaining electroneutrality in the digestive vacuole, which is essential for managing the acidic environment within this organelle.*

*Overall, this is a well-written, topical, and compelling study that significantly advances our understanding of the biology of the digestive vacuole and its role in P. falciparum. However, several aspects require clarification or further consideration:*

We thank the reviewer for their accurate description of the work, it’s importance to understanding parasite biology, and their positive comments about the manuscript.

*Queries and Suggestions for Improvement*

*1. Integrity of the Digestive Vacuoles*

*The experiments rely on the assumption that the digestive vacuolar membrane remains intact during isolation. While the authors use the movement of hemozoin crystals as an indicator of vacuolar integrity, this should be substantiated with a more robust assay. For example, the authors could measure the uptake of tritiated water and [¹⁴C] or tritiated inulin to determine the membrane's integrity quantitatively. Such an assay would strengthen confidence in the physiological relevance of their findings.*

This question is key to interpretation of cell- and organelle-attached patch-clamp recordings, which measure current flow through single ion channel molecules. In fact, these experiments do *NOT* rely on the assumption that the membrane remains intact. For example, one variant, the excised patch configuration, involves continued recordings after pulling the membrane patch away from the cell, which intentionally compromises membrane integrity to allow perfusion of the channel inner face. (See Box 2 of PMID: 33640269 for further description of this configuration.)

At the same time, we agree that retention of DV contents during harvest and recording is relevant to understanding how BVAC functions under physiological conditions within the living intracellular malaria parasite. Macroscopic uptake studies, such as the suggested tracer uptake experiments, are unfortunately not suitable because harvested DVs are contaminated with intact cells and organelles in our protocol and those of others. We prioritized speed of harvest to ensure intact metabolic processes over purity because a key advantage of patch-clamp is the ability to select individual cells or organelles for study, avoiding the need for highly pure preparations.

To address this question and add to our study’s physiological relevance, we have now performed and added S2 Fig. Here, we imaged DVs harvested using our protocol from a CRISPR/Cas9 transfectant line that expresses plasmepsin II with a c-terminal E(1)GFP. Because plasmepsin II-E(1)GFP is a soluble protein targeted to the DV, retained fluorescence in these DVs indicates preserved DV integrity throughout our enrichment method. See “E(1)GFP-tagged plasmepsin II, … (S2 Fig).”, lines 78-80 of the Results, “DVs harvested from the plasmepsin II-E(1)GFP …”, lines 395-402 and “plasmepsin II-E(1)GFP, a soluble DV reporter line”, lines 424-425 of Methods, and the revised S1 Table, describing the production of this transfectant and how the finding supports preserved DV integrity.

Notably, we selected the E(1)GFP variant because it was engineered to permit ratiometric pH measurements in mammalian endosomes and track acidification in nascent lysosomes. We envisioned tracking DV pH with this reporter. Unfortunately, our studies revealed that this reporter does not respond to changes in DV pH, presumably due to denaturation by pH ≤ 5.0 (described in ref. # 56 added to the revised paper) or partial digestion by DV-specific acidic proteases. See “Although the E(1)GFP …organelle”, lines 399-402.

*2. Relevant References*

*o	To provide a more comprehensive context for their results, the authors should cite article PMID: 35867395, which describes a conditional PfCRT knockdown mutant and its physiological implications.*

*o	In discussing PfMDR1 and its role in drug resistance, reference to https://doi.org/10.1371/journal.pbio.3001616 is necessary.*

*o	Additionally, when addressing digestive vacuolar acidification, the authors should include PMID: 37463201 in their references. These citations would strengthen the manuscript's connection to relevant prior work.*

These are relevant citations and have been added as references #29, #33, and #23, respectively.

*3. Molecular Identity of the Anion Conductance*

*While the study robustly demonstrates the existence of an anion conductance through electrophysiological evidence, its molecular identity remains unknown. The reviewer recognizes that full genetic characterization is beyond the scope of this work. However, given the existence of proteomic studies on the digestive vacuole (e.g., PMID: 21136929 and* *PMID: 28784333), it would be valuable for the authors to suggest potential candidates or provide a discussion of likely proteins involved. This would enrich the manuscript and offer a starting point for future investigations.*

We agree that gene identification will be stimulated by the discovery of this large conductance anion channel on the DV; the candidate gene strategy we developed has excellent potential for uncovering the channel’s molecular basis.

We have added a sentence about the DV proteome (PMID: 21136929, now cited as Ref #48), pointing out that there are several hypothetical multipass membrane proteins in that dataset (lines 286-288). We do not think it is appropriate to single out specific candidates as it would be overly speculative. Of note, we are pursuing some of these and other candidates identified through our own analyses, but that work is still in progress.

The other paper referenced by the reviewer (PMID: 28784333) describes a parasitophorous vacuole proteome and is not relevant to this submission.

*By addressing these points, the manuscript would provide an even stronger foundation for understanding the role of anion conductance in P. falciparum biology and its potential as a target for antimalarial drug development.*

We thank the reviewer for their constructive comments, which have strengthened our conclusions and improved the work’s presentation in the framework of prior DV studies.

------------------------------------

**Reviewer #2:**

*Saggu et al. "A large anion channel for malaria parasite digestive vacuole acidification and amino acid transport" establishes of a chloride channel in the malaria parasite digestive vacuole (DV) membrane. With great skill the authors perform patch clamp on the vacuole to characterize channel activity on the organelle's membrane. They go on to show voltage-clamp current recordings consistent with a single channel type. By varying the ion composition of the bath and pipette buffers the authors establish that the channel preferentially conducts anions up to the size of an amino acid. The authors go on to demonstrate that the channel is found in at least 3 commonly used strains of P. falciparum and that known transporters at the DV are not the cause of the channel activity, the channel is also insensitive to a range of known anti-malarials. While the channel properties are consistent with those of anion channels of other organisms, known channel inhibitors have no significant effect on the channel's activity.*

*The paper is a significant contribution presenting a to the community of malaria and ion channel research, adding high resolution functional (electrophysiology) data that explains Plasmodium spp. digestive vacuole physiology. The electrophysiological characterization of a novel organellar channel is a great achievement. I recommend the work to be published and made accessible to the community*.

We thank the reviewer for the accurate summary of the work and the strong positive comments about the work’s contribution to understanding DV physiology.

*I have one larger and few smaller concerns that I hope can help with work and that the authors may want to address.*

*My one major concern is about the recording buffer. In the methods Na-K buffer is mentioned to contain 70 mM KCl, 70 mM NaCl, 2 mM CaCl2, 2.5 mM MgCl2, 10 mM HEPES, 10 mM MES, pH 7.4, for modifications only major salts are mentioned. It is unclear if Ca and Mg are always present. For channel activity most relevant and interesting would likely be pH and Ca++ (see for example Riederer et al 2023). As there is a large pore in the DV membrane it appears the DV lumen would be clamped to the bath pH and [Ca++]. pH sets the protonation of the protein domains in the lumen which is relevant for proper protein activity, while pH and Calcium appear most relevant for regulation of channels in lysosomal compartments. I would expect the phycological pH of the DV lumen to be ~5 and Ca++ ~0.5mM. It would be helpful to see the physiology of the recording conditions discussed. If the researchers can add recordings, it would be most valuable to probe channel activity at low pH, while it can be interesting to also alter Ca++.*

Potential regulation of the DV channel by ions and other solutes is an important question. The Riederer *et al* 2023 paper (PMID: 36151054) discusses channel regulation in mammalian lysosomes. This review summarizes a large body of lysosomal studies that used **whole-organelle patch-clamp**, where the internal solution of the lysosome is replaced by the pipette solution, to characterize regulation of those mammalian channels by **luminal** pH and cytosolic [Ca^++^]. To achieve the whole-organelle patch-clamp configuration, these studies relied on vacuolin, a chemical that enlarges mammalian lysosomes by unknown mechanisms. We were unable to use vacuolin as it does not enlarge parasite DVs. Because ours is also the first direct transport study of DV conductances, caution also dictated that it is better to avoid chemical reagents with unknown mechanisms. As stated in the paper, our attempts to obtain reproducible whole-DV patch-clamp have been unsuccessful to date.

It is not correct that BVAC will clamp the DV lumen to the bath pH and [Ca^++^] values because 1) many studies establish the DV luminal pH is ~ 2.0 units lower than parasite cytosol (e.g. PMID: 6391917, 12427765, 17696875, 17381432) and that Ca^++^ also appears to be highly sequestered in this organelle (PMID: 12740366) and 2) our single-channel recordings indicate exclusion of cations with at least a 2.6-fold preference for anions. As stated in the manuscript, “BVAC likely excludes H^+^ … at diverse membranes”, lines 257-260.

Because the DV is not clamped to the bath pH and [Ca^++^], our organelle-attached patch-clamp cannot directly and precisely determine channel regulation by **luminal** ions. We are working to develop reliable whole-DV patch-clamp, which would enable direct examination.

Although our current technologies limit direct study of luminal composition effects, we have performed and added a few relevant experiments that provide useful early insights (new Figs. S6, S7, S8).

To examine regulation by luminal pH, we performed patch-clamp using DVs treated with both concanamycin A and NH_4_Cl (new S6 Fig). Concanamycin A inhibits the V-type ATPase on the DV, raising luminal pH (PMID: 37463201, added as ref# 23). NH_4_Cl is a weak base that dramatically raises DV luminal pH (PMID: 3905824, ref# 24) because of high permeability of membranes to NH_3_. These new references showed increased DV pH in both intact cells and freed DV preparations. This combination ensured a significantly alkalinized DV in our experiment. S6 Figure shows no significant effect on single channel gating, conductance, or open probability, suggesting that BVAC is not regulated by DV luminal pH. See “As several lysosomal … luminal pH.” Lines 154-162.

We have also now successfully obtained recordings with altered pH in the pipette solution to explore pH effects on the channel’s cytoplasmic face (new S7 Fig). Here, we found marked changes in gating and conductance, with reduced gating at pH 6.0 (S7A-C, left panels). At pH 8.5 (S7A-C, right panels), conductance was increased with a preponderance of functional dimer formation as observed occasionally at physiological pH (shown previously in S3D Fig.)

See “These pH studies establish H^+^-titratable domains on the channel, as expected for voltage-dependent proteaceous pores. They also distinguish the parasite DV channel from mammalian lysosomal channels, many of which are regulated by luminal pH [22]. As changes in cytosolic pH have a greater effect on BVAC behavior than luminal pH changes, these findings suggest greater exposure of H^+^-titratable residues that the cytosolic surface, possibly as a result of evolution to limit acid denaturation and protease digestion at the luminal channel face.”, lines 168-173.

We have now also performed patch-clamp using nominally Ca^++^-free patch-clamp solutions (solution M in S2 Table) to examine regulation by cytosolic Ca^++^, as seen with lysosomal BK channels. These studies revealed unchanged gating, conductance and open probability (S8 Fig). See lines “We also performed … ion channels.” Lines 174-178.

*Further concerns in order as they appear in the text:*

*I don't think the mechanism for hemozoin tumbling is known and should maybe not be promoted as indication of healthy DV function until then. My personal impression is that the movement depends strongly on light exposure.*

Although the mechanism of hemozoin tumbling is unknown, it has been used as a marker of freshly harvested DVs by several groups (refs. 4 and 17 of the paper); we also confirm the validity of this marker with Supplementary Videos 1-3 showing tumbling with freshly harvested DVs and loss of tumbling after ~ 1 h post-harvest. We use this only as a marker of freshly harvested DVs and an indicator of some ongoing metabolism; such use does not depend on mechanistic understanding. We are not aware of any published report suggesting an effect of light exposure and have not seen such an effect in our studies. We agree that cautious presentation is best and have added “Notably, because hemozoin tumbling occurs by an unknown mechanism, the link between fresh DV harvest and sustained metabolic activity in our experiments remains circumstantial.” Lines 88-90.

To add independent evidence on this question, we have now performed and added new experiments showing that the harvested DVs retain soluble plasmepsin II-E(1)GFP (new S2 Fig), demonstrating preserved DV integrity throughout our method of DV harvest. See “E(1)GFP-tagged plasmepsin II, … (S2 Fig).”, lines 78-80, “DVs harvested from the plasmepsin II-E(1)GFP …”, lines 396-397 and “plasmepsin II-E(1GFP), a soluble DV reporter line”, lines 425-426 of Methods, and the revised S1 Table, describing production of this transfectant and how the finding supports preserved DV integrity.

*The method to harvest DVs without detergent seems missing.*

We apologize for not clearly stating that the detergent-free harvest is performed identically, but without saponin addition to lyse infected cells. We have added “When used, … without pre-lysis in HBS with saponin.” Lines 336-337.

*Exact buffer compositions (as mentioned above) and temperatures at which the experiments where performed seem not given but are necessary to understand the data.*

We apologize for this oversight and have added a Supplementary Table with all recording solutions itemized (new S2 Table). We also clarify bath and pipette solution compositions in each Figure legend through reference to specific buffers in S2 Table.

All patch-clamp experiments were performed at room temperature, as added in Line 349-350.

*The calculations for the reversal potentials and permeability ratios should be given. Assuming room temperature for the experiments, I calculated P_Cl/P_cat = 2.2 a little lower than what is reported. I am somewhat surprised that no chloride was present in the pipette determining P_Cl/P_cat. The access resistance must have been very high without charge carriers, and a AgCl electrode would also produce an indeterminate voltage offset without use of a salt bridge in absence of chloride. Is sorbitol the only solute in the pipette, no other salts are present? It would perhaps be most unambiguous to provide all electrophysiology buffer conditions in a supplementary table.*

Our unfortunate omission of the full composition of the Fig. 2B pipette solution (sorbitol as the primary osmoticant) prevented the reviewer’s confirmation of our *P_Cl_/P_cation_* calculation from measured *E_rev_* values. As the reviewer correctly points out, our sorbitol pipette solution contained low Cl^-^ concentrations to permit stable recordings with the Ag:AgCl electrode in the pipette holder. This solution, Buffer D as provided in the new S2 Table, has 14 mM K^+^ and 9 mM Cl^-^, sufficient to prevent electrode drift. These values yield the *P_Cl_/P_cation_* of 2.6. We have clarified these points and added the equation (Eq. 3) used to estimate *P_Cl_/P_cation_* from *E_rev_* measurements to the Methods, “Differing … definitions.” Lines 374-381.

*Glutamate is a relatively large ion that may reduce buffer conductivity. It may be that the lower glutamate pore conductance scales with the buffer conductivity and can be rationalized in that way.*

It is not correct to assume that ion mobility and conductivity increase with ion size. For example, the large HEPES^-^ anion has a mobility similar to those of Na^+^ and of HCO_3_^-^ (*Water Resour. Res* 47:W04509, 2011); Group IA cations have mobilities that increase (not decrease) with size: Li^+^ < Na^+^ < K^+^ < Rb^+^ ≈ Cs^+^ (PMID: 27879836).

More fundamentally, the *E_rev_* shifted to -17.5 mV in this experiment, which used 140 K-glutamate and 140 KCl as the primary charge carriers in pipette and bath solutions (Buffers E and F in S2 Table, respectively). This change in *E_rev_* is a **thermodynamic**, not a kinetic measurement. It is therefore independent of ion mobilities and, instead, reflects absolute differences in permeability. From this *E_rev_* and Eq. 3 of the paper, we estimate *P_Cl_/P_glutamate_* of 2.1. We have revised the manuscript to clarify this point, “a negative *E_rev_* … for this channel.” Lines 145-146.

*It would be helpful to indicate 0 pA levels on the current traces in the figures to estimate if the channel fully closes or remains partially open. It would be helpful if the authors can comment on full/partial closure as well. Alternatively, some raw current traces can by nice as supplement.*

By convention, single-channel recordings are shown with open and closed levels marked and generally do not indicate 0 pA levels. This is because it is usually not possible to distinguish a channel with a constitutive partially open state from leak currents through the seal, which are always nonzero in real-world patch-clamp studies. A simplified equivalent circuit illustrates this:

 In this graphic, *R_a_*_,_ *R_m_*_,_ *R_s_*_,_ and *R_e_* represent pipette access resistance, resistance of the membrane patch under study, the seal resistance that produces leak currents, and the exit resistance for the rest of the organelle. *R_m_* has a value that decreases and increases with channel opening and closing, respectively. If there no active channels in the patch (or if channels are fully closed), *R_m_* >> *R_s_* and the deviation from zero current can, in most cases, be confidently attributed to leak currents through *R_s_*. A constitutively partial open channel state, as proposed by the reviewer would be indistinguishable from such leak currents in nearly all experiments. Such states have not been reported to our knowledge, but could be implicated if large numbers of recordings reveal that the apparent *R_s_* is systematically lower in channel-containing patches or through pharmacological or selectivity studies. Our studies over a 7-year period with two patch-clampers in the lab have not suggested this or other channel effects on the measured *R_s_*.

We agree that showing larger datasets of raw traces would be useful as it will more clearly demonstrate the channel’s complex and variable gating as well as establish fidelity of recordings. (Our recent review of parasite patch-clamp, PMID: 33640269 (ref# 18), also recommended this.) We have therefore added S4 Fig, showing 33.25 s of recording at a *V_p_* of ‑60 mV from consecutive traces recorded on a single BVAC molecule. Examination of individual traces in this Figure reveals the channel’s complex gating with subconductance levels, bursts of openings, periods where the channel is either primarily closed or primarily open, and periods with altered event durations. See “Examination of consecutively… DV membranes, [18]”, lines 110-114.

*Supplementary figures S2A, S2B, S6G and S7 are difficult to interpret without quantification. If there is enough data for i/i_max vs V (p_open vs V), single channel conductance and dwell times it would be good to show. Especially for the inhibitors, in Fig S7 it is difficult to make out that they are truly the same. To my eye it seems that with DIDS and phloridzin the channel flickers faster?*

We have the required data but do not see value in performing every one of the analyses for all control experiments as it would add excessive redundancy to the manuscript. We attempted to tailor our analyses for each experiment to allow conservative and confident interpretations, based on the hypothesis being tested. Notably, given the complex channel behavior, these analyses require manual examination of recordings and verification of script outputs, typically requiring 4-6 h of hands-on time to produce a single *i/i_max_* - *V_p_* plot, for example. Although we have developed and described scripts for dwell time analysis (PMID: 17292059), these analyses are not suitable for BVAC recordings as they impose two states on the channel (open and closed) and do not permit inclusion of subconductance levels.

Given these complexities, it is important to be selective about the types of analyses performed for each experiment. S2A Fig (now S3A) is a control experiment that excludes detergent artifacts; visual examination of traces is sufficient in this case because a detergent-induced artifact would be completely absent when saponin is eliminated. S2B Fig (now S3B) shows that ATP, GTP and phosphocreatine are not essential energy sources for BVAC gating and transport (the WOS additive, new S2 Table); detection of channel activity in patch-clamp without these energy sources indicates they are not essential. S6G Fig (now S11G) shows conditional knockdown of PfMDR1 in the 3D7 parasite strain; because we excluded a PfMDR1 contribution to BVAC formation (Figs. 3I and S11H) and we have already included IVs and *i/i_max_* - *V_p_* plots for the 3D7 strain (Figs. S5B-C), it is difficult to justify the hands-on time required for these redundant plots.

Our pharmacological studies used each candidate inhibitor at high concentrations to detect even weak levels of BVAC inhibition. As described in the manuscript, we selected a *V_p_* of -20 mV for sensitive detection of inhibitory effects because the channel is primarily open at this voltage. Each inhibitor was tested at concentrations well above their physiologically relevant levels; for example, chloroquine, mefloquine and artemisinin were used at 1 µM, at least 5x higher than their *IC_50_* values for the Dd2 strain used here (see PMID: 22949525 for these *IC_50_* values in our studies). The *P_o_* measurements, shown in Fig. 4, were tallied from a total of 1.8 – 8.0 seconds of single channel recordings; we also included *P_o_* values for each molecule (symbols superimposed on the bar graph in Fig. 4). These data adequately exclude physiologically significant BVAC inhibition by each of the inhibitors tested.

We included individual traces in S7 Fig. to establish that there are not dramatic changes in channel gating at a larger negative *V_p_* of -60 mV. Given the channel’s variable gating (apparent in the new S4 Fig., as suggested by this reviewer), we caution against attempting to decide if these traces “are truly the same”. The brief bursts of fast flickering seen in the DIDS and phloridzin traces can also be found in some traces of the inhibitor-free molecule shown in the new S4 Fig, recorded at an identical *V_p_* of -60 mV.

We have revised the Results to clarify that each inhibitor shown in Fig. 4 was tested at high concentrations to evaluate physiologically relevant block, “Each of these compounds … physiology”, lines 246-248. We acknowledge that it is difficult to exclude weak block by high concentrations of a given inhibitor as that might produce fast flickering behavior superimposed on the channel’s already complex gating. To present our observations cautiously, we added, “but weak interactions… our studies”, lines 249-250.

We thank this reviewer for their careful and constructive comments, which have strengthened the paper significantly through encouraging us to perform BVAC regulation studies, addition of all solution compositions, and clarification of several issues.
