# Supplementary material for: Identification of a large anion channel required for digestive vacuole acidification and amino acid export in Plasmodium falciparum
Source: PLoS Biol. 2025 May 30;23(5):e3003202. doi: 10.1371/journal.pbio.3003202 (PMC12158007; doi:10.1371/journal.pbio.3003202)
Supplement: Attachment — Submitted filename: Response to Reviewers 042925.docx [file pbio.3003202.s023.docx]

Itemized changes in response to reviewer comments.

We thank the editor and reviewers for their positive comments. We have itemized the responses and changes made below.

Reviewer #1:

*The reviewer thanks the authors for the constructive scientific dialogue. The authors have address my comments in full. No further suggestions.*

Reviewer #2:

*The authors thoroughly worked on the comments, and it is great to see the some of the questions addressed with new data. I appreciate the reporting of the experimental recording conditions and the availability of current traces.*

*Naturally I would have addressed some issues differently. While I appreciate the attempt to control pH I will argue that the results are not interpretable with the available data. The lack of pH control/quantification introduce a new major weakness that could be addressed either by weakening the conclusions or new data.*

*It is interesting that the E(1)GFP doesn't seem to respond to pH. I didn't find the protocol described to calibrate pH so I can't evaluate the technical side. I would think that it is unusual that the fluorescence is intact while the pH sensitivity is lost, rather I'd expect the fluorescence to be lost entirely. It is unfortunate the pH sensor isn't working as one could envision many experiments to explore pH homeostasis of the DV with a working pH sensor. In any case, it could be worthwhile switching to a different pH sensor for the reasons outlined below.*

In contrast to predictions, the original description of E(1)GFP indeed reports sustained fluorescence without pH responsiveness after protein unfolding at pH < 5.0 (see Fig. 1 of PMID: 19034435). While another pH sensor could allow measurement of DV luminal pH changes resulting from pharmacological treatments, more quantitative and precise studies of possible regulation by pH, ions, and other modulators will only be possible when other patch-clamp configurations are optimized for DV study. We have added “Future studies implementing the whole-organelle or excised patch configurations will permit more rigorous examination of luminal pH regulation as these methods allow precise control of pH at both channel faces.”, lines174-176.

*I agree with the authors response to reviewer 1's comment on the intactness of the DV. Since all recordings are performed in an attached configuration, it doesn't matter if the membrane, as a whole, is intact (as long as a patch can seal and the channels in the patch can reasonably expected to be folded well). On the flip side, I don't quite understand why the authors insist that the ion concentration of the DV interior would be unaffected by the bath ions. The channel never seems to fully close and is able to permeate all tested ions. Some of the recordings were done in presence of ATP, GTP, phosphocreatine which is theoretically helping to maintain low pH in the DV but without ATP (or the other energy source) I don't see how the DV would hold any ionic gradient.*

We thank the reviewer for concurrence on DV intactness. There are few other points made here for clarification.

We agree, of course, that ion contents in the DV can be affected by changes in bath solution composition. However, transport of H^+^ and other cations is limited by their permeability through BVAC. While single-channel patch-clamp cannot be used to exclude low-level permeability of cations, our work indicates that *P_Cl_* /*P_cation_* is least 2.6 (Fig. 2B and associated text). This does not mean that cations are measurably permeant.

Many studies have established that the DV membrane maintains a >100-fold H^+^ gradient within infected cells. This is maintained after DV isolation or permeabilization of infected cells to remove parasite cytosol. PMID: 12427765 (now added as ref. #23) used saponin and digitonin to permeabilize the infected cell membrane and the parasite plasma membrane, exposing the DV membrane to external medium; Fig. 8 in that paper shows that the DV remains acidic and that concanamycin A produces a rapid and large alkalinization. PMID: 36101540 performed similar studies with other chemicals that alkalinize the DV. PMID: 15811526 (added as ref. #24) performed biochemical studies with isolated DVs using a longer and more involved DV harvest protocol than ours, but still found the V-ATPase H^+^ pump remained active and responsive to inhibitors. To clarify this point, we have added “The DV maintains a large outward H^+^ gradient through the action of two H^+^ pumps in both intact infected cells and after separation from parasite cytosol [23, 24]; such gradients are only possible with negligible BVAC permeability to H^+^ because this channel’s large conductance would overwhelm the much lower rates of active extrusion pumps.” Lines 165-169.

Because active pumps have much lower transport rates, these gradients would only be possible if BVAC has negligible H^+^ permeability as we clarify here: Our BVAC conductance measurements indicate that Cl^-^ moves through a single open channel at a rate of 10^8^ ions/second (calculated from Faraday’s constant, where 1 pA corresponds to 6.3 x 10^6^ ions/second). By comparison, the primary DV H^+^ pump (V-type ATPase) pumps ~ 300 ions/second (based on related pumps, PMID: 21426606, new ref. #44). The nearly 1 million-fold greater flux through this channel indicates that any measurable H^+^ flux through BVAC would abolish the pH gradient across the DV membrane in both living cells and isolated DVs. Thus, observed pH gradients at this membrane indicate that BVAC must be impermeant to H^+^. Technical limitations on single-channel patch-clamp prevented us from formally excluding H^+^ flux, but this does not mean that the channel is permeant to H^+^. Similar reasoning indicates that BVAC Ca^++^ permeability must be very low because DVs maintain a Ca^++^ gradient through the action of inhibitor-sensitive ATPase pumps (PMID: 12740366, added as ref #47). To clarify this point, we have added “Although we established … . BVAC must exclude H^+^ because measured single channel currents are 10^6^ -fold greater than H^+^ pump rates [44]; even a low H^+^ permeability (*e.g. P_H+_*/*P*_Cl-_ of 0.01) would abolish the pH gradient across the DV membrane, compromising the DV acidic environment, hemoglobin digestion and heme detoxification. … negligible Ca^++^ flux through BVAC.” Lines 271-279.

Our data does not support the reviewer’s assertion that the “channel never fully closes”. The reviewer may have misinterpreted our prior response, where we stated that cell-attached patch-clamp cannot distinguish channels that remain open from leak currents through the pipette’s seal on the membrane. To clarify this point and establish that BVAC does fully close, we have now added a raw trace showing the response to a voltage step (S3A Fig., as requested by the reviewer below). See also “The combination of high-resistance seals on the DV membrane and the channel’s large conductance permitted confident identification of open and closed channel levels (S3A Fig).” Lines 96-98.

*A working pH sensor would help understand any potential pH dependent gating mechanisms in a well controlled manner. I am not sure the right papers are cited to argue for intact pH homeostasis of the isolated vacuole and the use of the employed compounds. I think PMID 37463201 measures cell proliferation in response to concanamycin and* *PMID 3905824 looks at digitonin released parasites but the DV is not isolated. NH4Cl only has an effect if the DV is acidic to begin with, similar for concanamycin. To my understanding, for the purpose of characterizing BVAC dependence on vacuolar pH, the authors are lacking a working pH sensor and can't assess the effect of the compounds used to alter the pH. Hence, I'd suggest to not interpret the results that rely on the presumed change of luminal pH.*

We have now added PMID: 12427765 (new Ref # 23), where the effect of concanamycin A is reproduced using DVs exposed to external medium by permeabilization of the host and parasite membranes (See Fig. 8 of that paper). As our method uses rapid harvest and avoids detergents used in that study, it is likely that DVs remain acidic in our experiments and that concanamycin A will produce the same rapid alkalinization. Notably, PMID 3905824 (our Ref. #26) used the same digitonin detergent to expose DVs to external medium, as evidenced by ready access of the DV to external ATP (Fig. 3 of that paper); the same figure establishes NH_4_Cl treatment alkalinizes the DV.

It is true that we were unable to quantify the luminal pH change in our study using single DVs. To present our findings more cautiously, we have added “We were, however, unable to quantify pH changes within single DVs. Future studies implementing the whole-organelle or excised patch configurations will permit more rigorous examination of luminal pH regulation as these methods allow precise control of pH at both channel faces.” Lines 173-176.

*Two minor points I want to ask to consider:*

*Ionic mobility (somewhat correlated to ion size) has an effect on conductivity. This can be used to scale conductivity for larger ions but also to correct for junction potentials. For general understanding somewhat relevant maybe is PMID: 7715244. I would love to talk to the authors about it. I feel we have some misunderstanding of each others' comments.*

It is indeed too bad that the peer review process doesn’t allow face-to-face discussions; I would also enjoy the discussion. Our prior statement about ionic mobility pointed out only that one cannot predict mobility from ion size. While ion mobility does affect conductance (i.e. the slope in our *i*-*V_p_* plots) and might yield an altered slope conductance, it would not alter our permeability estimates because these calculations are based on *E_rev_* (the *x-*axis intercept of this plot), not the slope. *E_rev_* is a thermodynamic, not a kinetic, measurement and does not depend on the rate of ion movement through the channel. To clarify this point, we have added “Because *E_rev_* is established at a voltage where infinitesimally small fluxes of the two ions are equal and opposite, this approach is a thermodynamic measurement not affected by the sizes or mobilities of the two ions.” Lines 149-151.

*For the 0 current indication, I understand that there will be leak currents. However, it would be informative to see if the channel fully closes. If there was a leak current proportional to the number of channels in the patch, one could assume this is due to only incomplete closure of the channel. Perhaps the right thing to ask for would be raw recordings from a voltage step protocol.*

We have added a raw recording showing the response to a voltage step protocol with a label for the zero current level, as requested (S3A Fig.). This recording establishes negligible leak currents and a fully closed channel as the closed channel current approximates the zero current level (red and blue dashes next to the current trace in S3A Fig. and “The leak current in this recording corresponds to a 30 gigaohm seal”, as added to the legend). The seal resistance of 30 gigaohm is considered a high value for any cell or organelle and is not consistent with incomplete closure of the channel. We also added “The combination of high-resistance seals on the DV membrane and the channel’s large conductance permitted confident identification of open and closed channel levels (S3A Fig).” lines 96-98.
